# Barriers and facilitators to extended working lives in Europe: a gender focus

**DOI:** 10.1186/s40985-017-0053-8

**Published:** 2017-01-31

**Authors:** Clare Ellen Edge, Anna Mary Cooper, Margaret Coffey

**Affiliations:** 1 University of Salford, Manchester, UK; 2 Department of Psychology and Public Health, School of Health Sciences, Allerton Building, Frederick Road, Salford, Manchester M6 6PU UK

**Keywords:** Age, Older workers, Women, Extended working lives, Facilitators, Barriers

## Abstract

**Background:**

There is a global imperative to respond to the challenge of a growing ‘old-age dependency ratio’ by ensuring the workforce is healthy enough to remain in work for longer. Currently more than half of older workers leave before the default retirement age, and in some countries (e.g. the UK), the time spent in retirement is increasing. At the same time across Europe, there is a gender employment gap, with 14.5% fewer female workers between 55–64 years old, and a large variation in the participation of older women in the workforce (ranging from 30–75%). As older women are under-represented in the workforce, increasing employment in this group has the propensity to go some way towards reducing the old-age dependency ratio to ensure continued economic growth.

**Objectives:**

This review explores the barriers and facilitators to extended working lives in Europe, particularly those that impact on women.

**Methods:**

A systematic mapping review process was undertaken using four electronic databases, MEDLINE, PsychoINFO, PsychEXTRA via Ovid and AgeLine via EBSCO, using the terms, ‘work’, ‘ageing’, ‘retirement’, ‘pension’, ‘old’, ‘barrier’, ‘extended working life’, ‘gender’ and ‘health and well-being’. Hand searching was also carried out in the *International Journal of Aging and Human Development* and the *International Journal of Aging and Society*.

**Results:**

The search resulted in 15 English language studies published from 1st January 2005 to the current date that met the inclusion criteria.

**Key findings:**

The key factors that influenced decisions to retire or extend working lives in Europe were health, social factors, workplace factors, and financial security and pension arrangements.

**Conclusions and implications of the key findings:**

Health was found to be the most commonly cited barrier to extended working lives in Europe, and a number of social inequalities to work exist by gender. Structural factors exist, such as the gender pay gap, which disadvantages women, while the nature of work itself differs by gender and can have a negative impact on health. Currently, women tend to exit the labour market earlier than men; however, changes in the state pension age are resulting in women being required to work for as long as men, in most countries. For women to remain healthy at work, workplaces need to consider a range of interventions, including flexible arrangements to both work and retirement to enable women to balance the demands of work with domestic and caring responsibilities that particularly impact on them.

## Introduction

People are healthier and live longer today than in previous years, while at the same time they have fewer children than they used to, leading to an increase in the ‘old-age dependency ratio’ [1]. This means a smaller proportion of working age people supporting a growing proportion of older citizens [2]. Data from Eurostat [3] indicates that life expectancy at age 65 has increased, and it is projected to continue to rise. As a result, most European Union member states are reforming their pension systems in order to improve the long-term sustainability of their public finances, together with adequate pensions [4, 5]. Generally, across Europe, these reforms have involved increasing the length of time that people spend at work before they can retire and are eligible to draw their pensions [6]. In addition, most European countries have implemented Active Labour Market Policies (ALMPs) aimed at extending working lives (EWL), and in addition, a number of countries (Belgium, Denmark, Finland, The Netherlands and Germany) have implemented flexible working policies [7]. The majority of changes to retirement age will take place in the 2020s. ‘Denmark, France, Germany and Spain have decided to raise the retirement age from 65 to 67 years, while the goal is 68 years in Britain and Ireland’ [6].

Table 1 highlights the key differences in statutory pension ages for males and females across 31 European countries (EU-28, plus the three European Economic Area (EEA) countries, Iceland, Lichtenstein and Norway), together with their respective gender pay and earning gaps.

**Table 1:** Statutory pension ages and gender pay/earning gaps

Country	Statutory pension age males (females)^a^	Gender pay gap^b, c^ (%)	The average gender overall earning gap^d, e^ (%)
Austria	65 (60)	22.9	46.7
Belgium	65	9.9	35.9
Bulgaria	64 years 4 months (61 years 4 months)	13.4	22.9
Croatia	65 (61 years 3 months)	15.4	23
Cyprus	65	6.5	33.7
Czech Republic	62 years 10 months (58–62)	22.1	41
Denmark	65 (67)	15.8	26.5
Estonia	63 (62 years 6 months)	28.3	32.2
Finland	63–68 (65)	18	27
France	65	15.3	32.9
Germany	65 years 3 months	21.6	45.3
Greece	67	15^f^	45.2^f^
Hungary	62 years 6 months	15.1	32.7
Iceland	67^g^	18.7	32.8
Ireland	66	14.4	34.7
Italy	66 years 3 months (63 years 9 months)	7.3	4.3
Latvia	62 years 6 months	15.2	16.1
Lichtenstein	64^h^	18^i^	^j^
Lithuania	63 years 2 months (61 years 4 months)	14.8	12.3
Luxembourg	65	8.6	38.4
Malta	62	4.5	56.3
Netherlands	65 years 3 months	16.2	49.1
Norway	62–75 (67)	14.9	34.4
Poland	65 years 7 months (60 years 7 months)	6.4	29.6
Portugal	66	14.5	27.8
Romania	65 (60)	10.1	29.9
Slovakia	62 (58 years 3–62 months)	21.1	37.5
Slovenia	64 years 4 months	2.9	12.8
Spain	65 years 3 months	18.8	38
Sweden	61–67 (65)	14.6	30.2
UK	65 (62 years 4 months)	18.3	47.6

Sources:

^a^Finnish Centre for Pensions (2015); http://www.etk.fi/en/the-pension-system-2/the-pension-system/international-comparison/retirement-ages/

^b^The gender pay gap is the difference in average gross hourly wage between men and women across the economy (the average gender pay gap in the EU is 16.1% based on the EU 28 provisional data; Ireland 2012 data) (Eurostat, 2013, 2016)

^c^Eurostat (2016); http://ec.europa.eu/eurostat/statistics-explained/index.php/Gender_pay_gap_statistics#Further_Eurostat_information

^d^The gender overall earning gap is the difference between the average annual earnings between women and men. It takes into account three types of disadvantages women face: lower hourly earnings, working fewer hours in paid jobs and lower employment rates (for example when interrupting a career to take care of children or relatives) (the average gender overall earning gap in the EU is 41.1%) (Eurostat, 2010)

^e^European commission (2015); http://ec.europa.eu/justice/gender-equality/document/index_en.htm#pay

^f^Eurostat (2010); http://ec.europa.eu/eurostat/statistics-explained/index.php/Gender_statistics

^g^OECD (2015); http://www.oecd-ilibrary.org/social-issues-migration-health/pensions-at-a-glance-2015/iceland_pension_glance-2015-58-en

^h^European commission (2015); http://ec.europa.eu/justice/discrimination/files/ad_2014_country_reports/2015-li-country_report_nd_final.pdf

^i^European commission (2013); http://ec.europa.eu/justice/gender-equality/files/epo_campaign/131218_epo_update_liechtenstein.pdf

^j^Data could not be located for Lichtenstein

The lowest retirement ages for women were found in Slovakia, Austria, Poland and Romania, while the highest were found in Denmark, Greece, Iceland and Norway [6]. Also highlighted is the gender pay gap, i.e. the difference in average gross hourly wages between men and women across the economy [8]. The latest figures from Eurostat (2016) [8] highlight an average gender pay gap of 16.1% (EU-28), which ranges from 2.9% in Slovenia to 28.3% in Estonia, with women generally earning significantly less. This pay gap has implications for retirement benefits, as the majority of state pensions are earnings related.

Work has been shown to be good for health across all ages [2, 9]. Specifically, ‘good quality’ work has been identified as vital for reducing health inequalities and a matter of social justice [2]. In addition, the workplace has been shown to be a potential ‘setting for health’, a concept first noted in the Ottawa Charter for Health Promotion [10] which asserted that the way populations organise work should create a healthy society. The long-term retirement has been shown to be detrimental to health, despite evidence of short-term health gains, particularly in respect of mental health [11, 12]. For example, retirement increases the likelihood of having at least one diagnosed physical illness by around 60% [13]. However, a recent systematic review has highlighted that much research has lacked detail of the interacting effects of subpopulations, such as by type of employment (for example, manual labour compared to office-based work), on health [14].

The European 2020 target is for 75% of the 20–64 year olds to be employed by 2020 [15]. In order to achieve this target, the under-represented categories of workers, which include younger and older workers, need to improve. However, in 2012, less than half of 55–64 year olds in the EU were working and more than half of older workers leave work before the default retirement age [16, 17]. This is despite the fact that life expectancy is higher and work is less physically demanding, which one would expect should led to higher, rather than lower retirement ages [18]. For example, in the UK, the average time spent in retirement is still increasing; since the late 1990s, it has risen from 20 to 22 years for men and 25 to 26 years for women [5], which highlights the complexity of the issue under review.

Looking at the participation in the labour market, there is a gender employment gap which widens through the life cycle, from 8.3 percentage points in the 20–29-year cohort to 14.5 percentage points in the older cohort (55–64 years) [19]. There is also large variation in participation rates of older women in the workforce, e.g. for the UK, they are 74.6% [20], while in parts of Europe (Malta, Slovenia, Greece, Poland and Italy), they are less than 30% [15]. As older women are an ‘under-represented’ category of worker, increasing the employment of this group has the propensity to go some way towards reducing the old-age dependency ratio [21], which is considered essential to ensure continued economic growth in the future [22].

Older workers are mainly employed in manufacturing (14%), human health and social work activities (11%), education (9%) and public administration (9%) and a further 21.6% are self-employed [17]. Changes in the demographic composition of older workers, aged 50–69, include a 21.8% rise of part-time workers in the EU-28 in 2012, compared to 17.0% for those aged 25–49 [23]. The greatest proportion increase in part-time work in percentage points between these age groups was in respect of women, from 29.9% (age 25–49) to 35.1% (age 50–69) across the EU-28 [23].

Given the economic imperative to maintain a healthy workforce and increase the number of older workers, this review will draw together the key barriers (including health) and facilitators to extending working lives, with a particular focus on gender.

## Methodology

### Review typology

The review method was informed by the framework suggested by Arksey and O’Malley [24] and used by Edwards and Brettle [25]. It followed the stages:

1. Identifying the research question
2. Identifying relevant studies
3. Study selection
4. Charting the data
5. Collating, summarising and reporting the results

A systematic mapping review process, designed to chart and categorise existing literature in order to identify the main themes and gaps [26], was used to draw out the key barriers and facilitators to EWL within Europe.

### Inclusion and exclusion criteria

The focus of this review was countries (*n* = 31) within the EU-28 and EEA. Articles were selected based on coverage of factors facilitating or inhibiting extending working lives (EWL). Papers were included from 2005, as a number of retirement policy directives across the EU and nationally have occurred specifically in the last 10 years.

### Search strategy

An initial scoping strategy was carried out using MEDLINE via Ovid in order to assess the literature available and to check the specificity and precision of the search strategy.

The search strategy was constructed around the three main concepts: barriers and facilitators, work and older workers. The search terms were age or ageing or aging, retire*, pension*, old*, barrier*, challenge* enhance*, facilitate* extended working life or lives, work* or job, occupation and employ*.

The following electronic databases were searched in August 2016: MEDLINE, PsychINFO, PsychEXTRA, MEDLINE(R) Epub Ahead of Print via Ovid and AgeLine via EBSCO. To support this, hand searching was carried out in the *International Journal of Aging and Human Development* and the *International Journal of Aging and Society*. In addition, the reference lists of included articles were scanned for any other articles that met the inclusion criteria.

### Study selection process

All citations were downloaded into an EndNote library, and duplicates were removed. Initially, the titles and abstracts were screened against the inclusion criteria to identify relevant studies by CEE and a proportion checked by AMC. Following this, the full texts were obtained for those papers marked as included and unsure, and these were screened to obtain the final included list.

### Process for charting the data

**Fig. 1:**
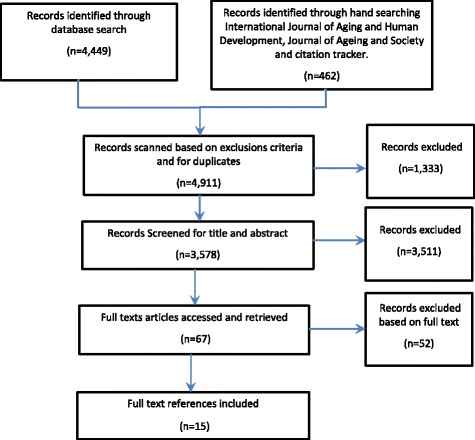
PRISMA flow diagram

This process was informed by the paper by Osei-Kwaqi et al. [27]. Following the identification of the included paper, factors relating to barriers and facilitators to EWL and those related to gender were themed into clusters and factors (Fig. 1).

### The key findings in relation to facilitators and barriers influencing people’s decision to retire or extend their working lives

The key characteristics of the 15 included studies in the review are presented in Table 2. Twelve of the studies were conducted in only one country: Norway *n* = 2 [28, 29], UK *n* = 4 [30–33], The Netherlands *n* = 2 [34, 35], Denmark *n* = 3 [36–38] and Germany *n* = 1 [39], with three of the studies being conducted across multiple countries [40–42]. In fourteen of the studies, the population was over the age of 40, with the exception of the Tuchsen et al.’s [37] study which looked at the increasing risk of female shift workers going onto a disability pension across all ages. Of the included papers, 11 were quantitative, of which eight were longitudinal [28, 29, 31, 35, 37, 38, 40, 41], three were cross-sectional [29, 36, 39] and four were qualitative [30, 32–34].

**Table 2:** The key study characteristics of included studies

	Author	Country	Study population	Design	Participants
[33]	Brown and Vickerstaff 2011	UK	Individuals approaching or entering retirement in the UK	Qualitative	*n* = 96 aged 50–65 year old (apart from 7 respondents slightly above or below the age brackets)
[42]	De Preter, van Looy and Mortelmans 2013	Austria, Germany, Sweden, Belgium, Spain, Italy, France, Denmark, Greece, Switzerland and The Netherlands.	Survey of Health, Ageing, and Retirement in Europe (SHARE) and macro data derived from the Organisation for Economic Cooperation and Development (OECD) and Eurostat	Quantitative-longitudinal	Total sample at t2 was *n* = 5,127 respondents, resulting in 440 retirement events. There were *n* = 2,582 men and *n* = 2,545 women in the sample
[40]	Hofaecker et al. 2016	Germany, UK (and Japan)	Survey of Health and Ageing in Europe (SHARE), English Longitudinal Study of Ageing (ELSA) and the Japanese Study of Health, Ageing and Retirement (JSTAR)	Quantitative-longitudinal	*n* = 5,172 English respondents aged 50 and over at t2 (*n* = 2,617 men, *n* = 2,555 women) *n* = 1,549 German respondents aged 50 and over at t2 (*n* = 909 men, *n* = 640 women) *n* = 892 Japanese older respondents at t2 (*n* = 468 men, *n* = 424 women)
[38]	Larsen 2008	Denmark	Danish survey of elderly individuals consisting of two waves from 1997 and 2002	Quantitative-longitudinal	*n* = 1,579 wage earners aged 52–57 years old at t2
[30]	Loretto and White 2006	UK	Workers from finance, education, local government, hospitality, caring services, professional occupations and the private sector were represented	Qualitative	Employees aged 50 and over *n* = 33, *n* = 19 men and *n* = 14 women participated across the four focus groups
[39]	Micheel, Roloff and Wickenheiser 2011	Germany	The data basis was constituted from the study ‘Continuing in employment in pensionable age’	Quantitative-cross sectional	*n* = 1,500 aged 55 to under 65 years old. This excluded self-employed individuals or those who were unemployed
[29]	Nicolaisen, Thorsen and Eriksen 2012	Norway	Drawn from the Norwegian Life Course, Aging and Generation (NorLAG) study	Quantitative-cross sectional	Workers aged 40–61 years (thinking about retirement) *n* = 2,339
[32]	Porcellato et al. 2010	UK	Individuals residing the North West of England	Qualitative	Age range 50–68 years old. Total sample was *n* = 56, of which *n* = 22 were in paid work (*n* = 31 males, *n* = 25 females)
[41]	Radl 2013	Austria, Belgium, Denmark, France, Germany, Greece, Italy, The Netherlands, Spain, Sweden and Switzerland.	SHARE survey data, which targets the European population	Quantitative-longitudinal	Aged over 50 years living in residential households and their partners. At t2 the total sample *n* = 12,154 (ranged from *n* = 407 Switzerland to *n* = 1,749 Sweden). Participants already retired *n* = 7,527 and still in work *n* = 4,627
[34]	Reeuwijk et al. 2013	The Netherlands	Participants were selected from the Study on Transitions in Employment, Ability and Motivation (STREAM).	Qualitative	*n* = 30 employees aged 58–64 years
[31]	Rice et al. 2011	UK	English Longitudinal Survey on Ageing (ELSA)	Quantitative-longitudinal	Individuals aged 50 years and over at baseline and at retirement age (age 60 years for women, 65 years for men) at 4-year follow-up, living in private households in England *n* = 1,693 at t2
[28]	Solem et al. 2016	Norway	Norwegian Study on Life Course, Ageing and Generation (NorLAG)	Quantitative-longitudinal	*n* = 2,401 employed workers aged 40–79 years. *n* = 605 were 57 years old and over at t2
[36]	Thorsen et al. 2012	Denmark	Danish National working Environment Survey (DANES)	Quantitative-cross sectional	Total sample *n* = 3,122 aged 50 years and over.
[37]	Tüchsen et al. 2008	Denmark	Danish Work Environment Cohort Study (DWECS)	Quantitative-longitudinal	Employees aged 18–59 years females *n* = 3,980, males *n* = 4,025 at t3
[35]	van Solinge and Henkens 2014	The Netherlands	Older employees residing in The Netherlands	Quantitative-longitudinal	*n* = 1,458 older workers at t2 aged 50–59 years old at baseline.

#### Factors facilitating or enabling extended working life

Through the systematic mapping exercise, four main clusters (each of which contained a number of factors) were identified that related to influences on people’s retirement decisions (which vary by occupation and gender) (Table 3):

**Table 3:** Systematic mapping of included studies

Cluster	Factor	Evidence	Study population	Study outcome	Gendered context
Health context	Ill health as a barrier to extending working life (EWL)	[33]	UK	Health was a key barrier to EWL. Mental health problems, arthritis and diabetes along with high blood pressure and angina were the key health problems in older workers.	No gendered differences were discussed in respect of health as a barrier to EWL, except that the type of roles women and men tend to adopt differ.
		[42]	Austria, Germany, Sweden, Belgium, Spain, Italy, France, Denmark, Greece, Switzerland and The Netherlands	Having one or more limitations regarding activities of daily living, in respect of physical health, predicted retirement.	In the multivariate analyses, it was shown that men tended to retire later than women.
		[40]	Germany, UK and Japan	There was a high incidence of retirement due to ill health in Germany.	In Germany, it was found that involuntary retirement (including for ill health) was more frequent among men than women, but this was not found in the UK (or Japan). Overall, females were shown to retire earlier than men.
		[38]	Denmark	Poor health reduced planned retirement age.	No gender differences were reported in relation to this variable.
		[30]	UK	Workplace stress and the negative effects on their health were cited as a key influence for with those who wished to retire as soon as possible.	Gender was not associated with this particular finding.
		[32]	UK	Ill health was seen as a key barrier to EWL.	There were a disproportionate number of male older workers who experienced mental ill health, particularly stress. Stress was reported to relate to changes to job conditions within the workplace, and changes in the job market, such as commuting and target driven roles.
		[31]	UK	Ill health was found to be a predictor of retirement. Specifically, older workers displaying depressive symptoms or mobility problems (leg pain in particular) were more likely to retire early.	Gender was adjusted for and it was found that women were more likely to retire before men.
		[28]	Norway	Retirement intentions were significantly related to retirement behaviour. Older workers with poor health often retire earlier than they prefer. Overall, amongt all groups (including those in good health), workers tended to retire earlier than they wish, as a result of fewer opportunities being available. Health promoting environments, which include training or adapting working conditions are encouraged.	The main finding concerning gender was that gender differences in retirement patterns were small. However, when controlling for confounders, e.g. income, type of work and education, male workers tended to retire earlier than female workers.
		[35]	The Netherlands	Good health was a predictor of EWL.	No gender differences were reported.
	The negative impact of work on health as a barrier to EWL	[33]	UK	Manual work and caring roles carry particular health burdens in older workers.	Males, particularly working class men, described physical impairments often linked to manual roles. Additionally, caring roles, which tend to be female dominated, were seen as a risk to health and well-being.
		[41]	Austria, Belgium, Denmark, France, Germany, Greece, Italy, the Netherlands, Spain, Sweden and Switzerland	Ill Health is an obstacle to EWL in Western Europe. Routine service and manual workers were shown to be more likely to retire involuntarily due to ill health.	A higher prevalence of involuntary retirement was found among men in intermediate occupations, as well as male skilled manual workers and farmers.
		[37]	Denmark	When controlling for variables including age, health and socio-economic status, an increased hazard for disability pension was found for females only.	Female shift workers had more chance of becoming recipients of disability pension after controlling for a number of variables including health and socio-economic status.
	Positive health benefits of EWL	[30]	UK	Remaining in work was reported to have positive health benefits, compared to retirement, which was considered unhealthy	Gender was not associated with this particular finding.
		[32]	UK	Good health and physical fitness were seen as facilitators to EWL.	No gender-based findings in respect of health were discussed.
	Subjective experience of health as a barrier to EWL: health pessimism	[33]	UK	A number of factors including perceived job satisfaction, pressures from work, caring obligations, financial pressures affected the subjective experience of health. Those in lower socio-economic groups tended to show health pessimism, which predicted retirement.	Women tended to adopt caring roles and additionally had more caring obligations outside of work than men.
Social factors	Social and leisure activities as a driver or barrier to EWL	[29]	Norway	Men whose hobbies included fishing and hunting retired early and women who took on volunteering roles were more likely to have a preference for EWL.	Leisure activity (such as fishing and hunting) was associated with a preference for early retirement in men. Women with volunteering roles were more likely to extend their working lives.
		[34]	The Netherlands	The desire to spend time away from work was cited as a factor pulling individuals towards retirement.	Only 20% of the sample were women, so these findings disproportionately reflect the views of men.
	Caring responsibilities as a barrier to EWL	[42]	Austria, Germany, Sweden, Belgium, Spain, Italy, France, Denmark, Greece, Switzerland and The Netherlands.	Caring for a grandchild was a predictor of retirement. Although caring for a dependent in the household predicted EWL.	There were no gender differences in the finding that caring for grandchildren had a positive effect on early retirement.
		[32]	UK	Caring responsibilities were highlighted as a key barrier to work.	No gender-based differences were discussed.
		[34]	The Netherlands	Caring for others was highlighted as a key barrier to work.	No gender-based differences were discussed: only 20% of the sample were women, so these findings disproportionately reflect the views of men.
	Education and employment level as a barrier/facilitator to EWL	[42]	Austria, Germany, Sweden, Belgium, Spain, Italy, France, Denmark, Greece, Switzerland and The Netherlands	Older workers with a higher education tend to work for longer.	The relationship with education applied to both males and females, although men retired later than women.
		[40]	Germany, UK and Japan	In Germany, those with lower educational levels were more likely to retire than those with a higher educational level.	In Germany, it was found that involuntary retirement (including for ill health) was more frequent among men than women, but this was not found in the UK or Japan. Overall, females were shown to retire earlier than men. In UK, women retired more for personal reasons than men.
		[38]	Denmark	Those with higher income, educational status and higher levels of vocational training were more likely to plan to EWL.	No gender differences were observed with educational status, but women only showed an increase in retirement age with increasing vocational training.
		[39]	Germany	Professional status and income as indicators of socio economic status were predictors of plans to EWL.	Both income and professional status played a more significant role for women than for men. Women with high professional roles show greater chances of plans to EWL than men.
		[32]	UK	Lack of educational qualifications prevented a number of participants from EWL by re-entering the job market. Many respondents regretted not achieving qualifications at an earlier age.	Both genders discussed a lack of educational experience as a barrier to competing in the job market.
		[28]	Norway	Significant relationships were found between retirement intentions and retirement behaviour. Older workers with low education often retire earlier than they prefer. Lower educational level and blue collar workers were more likely to retire earlier than more highly education workers and white collar workers, possibly due to a lack of opportunity to remain in work.	Few gender differences were found but when controlling for confounders male workers tended to exit the labour marker earlier than female workers.
	Social class	[41]	Austria, Belgium, Denmark, France, Germany, Greece, Italy, the Netherlands, Spain, Sweden and Switzerland	Social class was shown to exert a strong influence on retirement, whereby those in lower social classes were more prone to involuntary retirement.	Gender differences in retirement behaviour appear to be largely driven by women’s lower class positions.
	Partner status	[39]	Germany	Married people are much less willing to EWL than unmarried people.	The differences by gender are slight and statistically insignificant.
		[29]	Norway	Single women showed a preference for retiring later than married women.	This result applied to females only.
		[31]	UK	Retirement of a partner, among other factors predicted retirement	Female gender, low retirement wealth and retirement of a partner, among other factors predict retirement.
		[35]	The Netherlands	Older workers who did not have a partner were more inclined to EWL than those who did.	No gender differences were reported.
	Negative social norms that act as barriers to EWL and internal beliefs about ageing as a barrier to EWL	[30]	UK	Participants in the study talked about the barriers they impose on themselves that act as a barrier to EWL, which were negatively affected by local and regional culture and pressure from retired friends.	The normative belief ‘male breadwinner role’ (p. 503) is entrenched in expectations of men’s retirement choices, although women also stated they were more likely to continue working for social as well as financial reasons.
		[32]	UK	A theme generated from this study was ‘negative perceptions of self: self-fulfilling prophecies’ (p.91). Although some positive attitudes were noted about older workers, participants expressed ageist social norms that they held, which constrained their opportunities to EWL.	Both men and women cited negative perceptions of self and ageist attitudes as barriers to EWL.
Workplace factors	Workplace conflict and poor work mentality of colleagues as a barrier to EWL	[34]	The Netherlands	Workplace conflict signalled early retirement. Poor work mentality of colleagues was also cited as a push factor for retirement.	The data provided in the study in respect of conflict related to a female, although only 20% of the sample were women so these findings disproportionately reflect the views of men.
	The social role of work a facilitator to EWL Volunteering role as a facilitator to EWL	[30]	UK	Enjoyment of work in relation to being around good people was cited as a reason for continuing working.	The social role is a particular facilitator to EWL in females (especially those in part-time work) in comparison to men.
		[29]	Norway	Females who took on voluntary work showed a preference for retiring later.	Women prefer different leisure activities such as volunteering, which signals a preference for EWL.
	Lack of choice and poor quality jobs available	[30]	UK	Participants cited concern about the choice and quality of jobs available to older people.	Employers were seen to hold a stereotypical belief that older women preferred part time work.
	High pressure/physically demanding jobs act as a barrier to EWL	[38]	Denmark	Job demands lowered planned retirement age and satisfaction with work hours, and the opportunity to use skills increased preference for EWL. Men who have poor job security plan to retire around half a year earlier than those who do not and being able to organise their own work was important to men but not women.	The job demands result applied to both men and women but 28% of women found their job demanding compared to just 18% of men. Poor job control and job security impacted negatively on men’s EWL only. Men are more influenced by the quality of job dimensions in their retirement planning.
		[30]	UK	Manual work was seen to cause increasing deteriorations in performance with age.	Older women in manual work were seen as been affected by deteriorations in performance more than men.
		[34]	The Netherlands	High pressure work and physical demanding jobs were cited as push factor towards retirement because they reduce the ability of older workers to EWL.	Only 20% of the sample were women, so these findings disproportionately reflect the views of men (in relation to high pressure work and physically demanding jobs and insufficient skill use).
		[35]	The Netherlands	Increasing job pressures were associated with earlier planned retirement but not actual retirement age.	No gender differences were reported.
	Lack of recognition, insufficient use of knowledge, skills and experience at work as a barrier to EWL	[30]	UK	The value of skills learnt by older workers was seen as largely ignored by employers.	No gender-based differences were reported in respect of this finding.
		[32]	UK	Insufficient use of knowledge and an unwillingness to train older workers to learn new skills was expressed by older workers.	Both men and women experienced poor quality jobs.
		[36]	Denmark	Perceived ‘ageism’, ‘lack of development possibilities’ and ‘lack of recognition’ were all significant for men but not for women (p. 441).	Lack of recognition as a cofounder was significant in men only.
	Recognition, sufficient use of knowledge, skills and experience at work as a facilitator to EWL	[38]	Denmark	The opportunity to use skills increased planned retirement age.	This result applied to both men and women.
	Organisational changes as a barrier to EWL	[32]	UK	Changes in the workplace caused participants to feel resistance and was an obstacle for older workers.	No gender differences were reported.
		[34]	The Netherlands	Organisational changes including restructuring and continuous changes in the way the job is done were found to push older workers towards early retirement.	Only 20% of the sample were women, so these findings disproportionately reflect the views of men.
	Rewarding work: making a positive contribution at work as a facilitator to EWL	[32]	UK	Making a positive contribution to the organisation was seen by many respondents as a driver for continuing working.	No gender differences were found in relation to this particular response, although women talked about social drivers to extending working life whereas men did not.
		[35]	The Netherlands	Higher levels of challenge at work were associated with an increasing chance of EWL.	No gender differences were reported.
	Negative or ageist attitudes act as a barrier to EWL	[30]	UK	Ageism in the workplace was identified by participants in relation being seen as an ‘easy target’ (p.500) in relation to redundancy and, generally, a lack of equal opportunity.	There were no particular differences found in relation to ageism.
		[32]	UK	Although older workers were shown to have positive views of themselves as ‘fitter, healthier, more capable and sustainable’ (p.92), this was incongruent with others’ views, which acted as a barrier to EWL.	No gendered differences were found in respect of ageist attitudes.
		[36]	Denmark	Perceived ‘ageism’ and ‘lack of recognition’ were all significant for men but not for women (p. 441).	Ageism in particular showed stronger associations in men with plans for retiring than females. Women were shown to retire earlier than males overall.
		[35]	The Netherlands	Workplace norms and supervisors’ attitudes shape older workers’ retirement intentions.	No gender differences were reported.
	Support from management to EWL	[35]	The Netherlands	Perceived support from management was positively associated with planned retirement age.	No gender differences were reported.
	Flexible conditions as a facilitator to EWL	[39]	Germany	Older workers had a preference for flexible working conditions such as reduced hours, working from home, control over hours as a facilitator to planned EWL.	No gender differences were cited for flexible working conditions.
	Working hours satisfaction as a facilitator to EWL	[38]	Denmark	Increasing working hours and working hours satisfaction increases planned retirement age.	The number of working hours increases retirement age for men, but there was no significant difference between men and women.
	Workplace size: smaller organisations are perceived as having more flexibility and choice to EWL	[40]	Germany, UK and Japan	Those in smaller organisations have more choice and more likely to EWL, although they tend to involuntary exit for reasons of ill health.	Organisation size impacted on both genders.
		[30]	UK	Larger organisations were viewed as being less willing to promote older workers.	Both men and women talked about workplace size.
		[39]	Germany	The smaller the organisation the more likely participants were willing continue employment post retirement age.	No gender differences were cited in respect of the organisation size.
	Lack of training and opportunities, development possibilities and career progression as a barrier to EWL	[30]	UK	Lack of development opportunities were cited by participants as a key barrier to EWL.	There was a stereotypical belief from employers that older women preferred part-time work.
		[32]	UK	Many participants felt that they lacked the skills needed to compete in the labour market, specifically IT skills.	Both males and females discussed a lack of development opportunities.
		[36]	Denmark	‘Lack of development possibilities’.	Lack of development possibilities acts as a barrier to EWL in men only.
		[35]	The Netherlands	Older workers who perceived more possible growth opportunities at baseline turned out to retire later than those who did not.	No gender differences were reported in respect of this finding.
	Workability: balancing the interplay between resources and demands as a facilitator to EWL	[36]	Denmark	Poor workability was a strong predictor of retirement for both men and women.	Poor workability predicted retirement in both genders.
Financial and pension arrangement	Financial disincentives exist for employers to recruit older workers	[32]	UK	Almost a quarter of participants put forward financial disincentives for employers to employ older workers, such as training costs and the need for costlier flexible hours with older workers.	Both genders talked about financial disincentives that exist for employers to recruit older workers.
	Finance as the main driver to continuing work and/or to supplement pension	[40]	Germany, UK and Japan	Social welfare has an impact on choice in respect of retirement when on a low income. Those with low education (across both genders) are disadvantaged in that they have to continue working for financial reasons.	There were no gendered differences to this finding.
		[30]	UK	For many respondents, the main driver to EWL was financial, reflecting a number of personal and family needs.	Both genders experience finance as a driver to continuing work.
		[39]	Germany	Willingness to EWL increases with lower monthly net household income.	Women with low household incomes show greater chances of plans to EWL than men.
	Pension wealth is a predictor of retirement	[38]	Denmark	Pension wealth was a predictor of earlier preferred retirement age in men.	Earnings were more important in predicting men’s than women’s retirement preference. A 10% income rise increased age of planned retirement by 0.331 years for men but only 0.044 years for women.
		[31]	UK	Individual finance, specifically pension wealth was a key driver of early retirement.	Gender was controlled for and women were more likely to exit the labour market than men.
	Financial opportunity to retire a push factor into retirement	[42]	Austria, Germany, Sweden, Belgium, Spain, Italy, France, Denmark, Greece, Switzerland and The Netherlands	High tax on continued work was a push factor in to retirement.	There were no gender differences in respect of this finding, although women tended to retire earlier than men.
		[34]	The Netherlands	The financial incentive to retire played an important role in retiring for all of the participants.	Only 20% of the sample were women, so these findings disproportionately reflect the views of men.

- Health
- Social factors
- Workplace factors
- Financial security and pension arrangements

The cluster ‘health context’ consisted of four factors: ill health as a barrier to EWL, the negative impact of work on health as a barrier to EWL, positive health benefits of EWLs and the subjective experience of health (health pessimism) as a barrier to EWL. Health was cited in the majority (*n* = 11) of the reviewed studies [28, 30–33, 35, 37, 38, 40–42]. Nine of these studies cited health as a barrier to EWL [28, 30–33, 35, 38, 40, 42] while two also noted that there were positive health benefits of EWL [30, 32] and one highlighted the qualitative experiences of the UK older workers’ subjective experience of health, which was affected by social context and individual circumstances such as financial constraints [33]. Shift work and manual work were found to negatively impact on health in three of the studies [33, 37, 41]. Eight studies cited gender differences in respect of health or retirement. Firstly, in respect of retirement, whereby females were shown to retire earlier than males across the study populations [31, 40, 42] with the exception of Norway [28] where males were shown to retire earlier, although this result was not consistent. Three studies specifically cited gender-based differences to health as a barrier to EWL; firstly, in respect of mental health and stress, whereby men were disproportionately affected compared to females [32], and secondly, in respect of involuntary retirement (including by ill health), whereby men were more prone to becoming involuntarily retired than females [40, 41]. There was also a gendered context to the impact of work on health, firstly in a study conducted in Denmark, which illustrated the negative impact of shift work on the health of older workers, specifically in females [37]. Secondly, Brown and Vickerstaff [33] in their qualitative UK study found that men, particularly working class men, tended to adopt manual roles that were linked to having physical impairments, while women tended to be in caring roles, which also carry their own health risks related to the emotional impact of their jobs.

Social factors related to retirement choices were identified as follows: social and leisure activities, caring responsibilities, education education/employment level, social class, partner status, negative social norms and internal beliefs about ageing that impact on retirement decisions.

The desire to spend time away from work, e.g. for leisure activities [29, 34] or to care for a grandchild [42] was found to be a barrier to EWL. Having caring obligations was also found to be a barrier [32, 34], although De Preter [42] found that caring for a dependent within the household predicted EWL. Educational or employment status was found to be both a barrier and a facilitator to EWL, and there were some intercountry differences [28, 32, 38–40, 42]. Older workers with higher education [38, 40, 42] and professional status [39] tended to work for longer. Older workers with low education often retire earlier than they prefer [28], and in the UK, a number of participants reported that their lack of qualifications, which they regretted, prevented them from competing in the job market, thereby acting as a barrier to EWL [32]. Similarly, social class (which can incorporate education level) was found [41] to exert a strong influence on retirement decisions. There were also some gender differences across studies and by country in respect of educational or employment level as a social barrier to EWL. Females were found to retire later than men in Norway [28] and earlier than men in Germany and in the UK [40]. Across Europe, gender differences in retirement were largely driven by women’s lower social class positions [41], and personal reasons were cited more so by women than men in the UK [40]. In terms of EWL, for women, an increase in retirement age was found with increasing vocational training [38], and women with high professional roles showed greater chances of having plans to EWL than men [39].

Partner status was also shown to impact on EWL, with single people appearing to be more willing than married people to EWL [29, 35, 39]. In addition, having a partner who is retiring was found to predict retirement in the UK [31].

In two qualitative UK studies, negative social norms and internal beliefs about ageing, specifically in relation to the roles that men tend to play as the ‘breadwinner’, were found to be a barrier to EWL [30, 32]. However, one study also found that women may want to stay in work for social as well as financial reasons [30].

A range of ‘workplace factors’ associated with retirement choices were identified in nine of the review papers [29, 30, 32, 34–36, 38–40]. Of these, facilitators to EWL were found to be positive social aspects of work, e.g. work colleagues [30], the nature of the job, e.g. volunteering [29], the opportunities to use/develop skills [35, 38], making a positive contribution to the organisation [32], higher challenges [35], having support from management [35], flexible working conditions, including working hours satisfaction [38, 39], and working in a smaller organisation [39, 40]. The key barriers to EWL were found to be workplace conflict and poor work mentality [34], lack of choice and poor quality of jobs available [30], high pressure/physically demanding jobs [30, 34, 38], lack of recognition and insufficient use of skills [30, 32, 36], organisational changes [32, 34], negative or ageist attitudes [30, 32, 36], with supervisors attitudes being found to shape retirement intentions in The Netherlands [35], organisational size, with larger organisations being viewed as less willing to promote older workers in the UK [30], lack of development opportunities in respect of career and skill progression [30, 32, 36], and poor workability, i.e. balancing the interplay between resources and demands [36].

Looking at ‘workplace factors’ in relation to the gendered context, in respect of the social role of work and volunteering as a facilitator to EWL, women tended to take up volunteering, which facilitated EWL [29]. Further, Loretto and White [30] found women felt the social aspect of work were facilitative to EWL, whereas men did not relay this. Ageism in the workplace was shown to have stronger associations with retirement in men than women [36]. A number of gender differences were also found relating to a range of confounding workplace factors such as development possibilities and recognition, which were significant for men but not for women [36]. Larsen [38] found that increasing working hours was a factor in increasing planned retirement age in men but not in women. Finally, in respect of choice of work available and physically demanding work, Loretto and White [30] found evidence of a stereotypical belief among employers that older females have a preference for part-time work and older workers talked about the perception that women in manual work were affected by deteriorations in performance at a faster rate than men.

Four factors were identified that related to ‘financial and pension arrangements’: financial disincentives exist for employers to recruit older workers, finance as the main driver to continuing work and/or to supplement pensions, pension wealth as a predictor of retirement and the financial opportunity to retire as a push factor into retirement. Financial factors found to facilitate EWL were the need to continue to earn money or supplement pensions for personal/family needs [30, 39, 40]. Conversely, barriers to EWL were financial disincentives for employers to employ older workers [32] having sufficient pension wealth [31, 38], high tax charges on earnings [42] and financial incentives to retire [34].

Financial factors were fairly consistent across gender (although women are shown to retire earlier) and countries apart from in two cases. Firstly, a study in the UK found that those with lower educational levels were more likely to need to work for financial reasons, whereas in Germany, the opposite was true as those in lower paid roles were more likely to retire [40]. While in Denmark, pension wealth was found to be a predictor of earlier preferred retirement age in men but not in women [38].

### Discussion of the key facilitators and barriers influencing people’s decision to retire or extend their working lives

#### Health

As identified in this review, health is the most frequently cited factor inhibiting EWL and healthier people are found to retire later, which corroborates previous reviews [36, 43, 44]. Mental health, arthritis, diabetes, blood pressure, angina and mobility difficulties have been identified as particular health issues for EWL in older workers. In Western Europe, it is estimated that approximately 30% of men and women aged 50–64 years need urgent adjustments at work because of health problems in order to prevent the risks of early retirement and work disability [16]. This review supports the idea that work itself can be damaging to health and adjustments are needed, in that shift work, manual work and caring roles have been shown to have a negative impact on health. The need for workplace adjustments has been found to be far higher than their actual implementation [45], suggesting that ‘occupational and public health services should put a strong emphasis on preventive measures in tackling work disability and subsequent early retirement’ [46]. In this regard, the workplace should be seen as a priority setting for health promotion [46] and policy measures should focus on the following:

1. Primary prevention by means of health promotion; reinforcing healthy lifestyles, counteracting unhealthy lifestyles and improving the physical work environment
2. Secondary prevention by early detection and treatment of chronic diseases
3. Tertiary prevention through supporting those affected by chronic disease through treatment and rehabilitation [47].

The notion that work itself impacts negatively on health echoes a previous UK policy review of workplace infrastructure [48]. In fact, Buckle [48] states that ‘there appears to be remarkably limited evidence from appropriately designed intervention studies that changing the infrastructure will enhance the well-being, the performance or increase the working life of older workers’. Similarly, a prior review of the literature on the effectiveness of interventions for ageing workers around EWL, work ability and productivity [49] found only limited evidence for various workplace programmes ranging from a 6-month vitality intervention with personal coaching to vocational rehabilitation activities. In the absence of strong evidence on older workers, McDermot [50] also carried out a prior review suggesting a number of interventions that *if* used with older workers *may* benefit their health, well-being and work ability across the life course. These included workplace physical activity interventions and those aimed at risk factors for chronic illness, e.g. smoking, diet and physical inactivity. Similarly, a review in the USA also suggested that workplace-based health and wellness programmes are the key to enabling older workers to extend their working lives [51]. Good practice identified by a review for the National Health Service in the UK for EWL [44] included sustainable lifestyle health promotion, implementing management standards for stress at work for both stress and non-stress based issues and other occupational health arrangements such as health monitoring for the over 40 workforce.

While the available evidence from previous reviews and the current review do not allow us to draw specific conclusions about how to improve the health of older women workers to extend working lives, given that they are more likely to be of low socio-economic status and in low paid part-time or temporary jobs, the measures outlined above to reduce work disability could have a proportionately more positive impact on females who are disproportionately clustered in these jobs. Moreover, the key employment-related policies aimed at combatting women’s poverty could subsequently improve their health and facilitate EWL. Such policies include the provision of well-paid parental leave (currently available in Denmark, Sweden and Slovenia) and the need to extend good quality childcare provision [52]. In addition, a recent UK report highlighted that support and awareness around the menopause was a key priority to actively address to facilitate a better environment at work for older women [53].

#### Social factors

Educational or employment level was identified in the current review as a barrier and a facilitator to EWL, as in previous reviews [43, 44]. This review has also supported policy evidence suggesting that there are a number of fundamental differences between women and men in terms of employment characteristics [54] and retirement decision-making [55]. For example, women’s work is more likely to be less secure, part-time, and undervalued [3]. Figures for Britain in 2013, showed that 42% of women were employed on a part-time basis in comparison with only 13% of men and women’s pay is also consistently lower than men [56]. Women over 50 have been long shown to have more discontinuous employment histories than men as a result of breaks associated with having children [3, 18]. This results in fewer chances to climb up the career ladder for women, which is closely related to pension entitlements and subsequent retirement choices.

In respect of the type of work that men and women carry out, women take on more ‘emotion work’ in caring roles. Although this review did not highlight gender differences in unpaid caring roles, an excluded study showed that women take on a significantly higher burden than men [57]. Good quality flexible working was identified in the review and is widely cited in the retirement literature as a mean of enabling older workers (particularly women) to spend time caring for others and maintain a satisfactory work-life balance [46, 58]. In order to mitigate the challenges of balancing paid work with domestic responsibilities, interventions that match working conditions with the reasons why individuals wish to retire early [29] are also recommended. In line with this, some researchers have argued that, due to increased heterogeneity among older workers, individually focused arrangements harnessing flexible employment arrangements will be of most benefit to the older worker [59].

Flexible or partial retirement policies are another potential option to help in the transition from work to retirement [17]. However, given the complicated interactions between domestic environments and gender roles, Loretto and Vickerstaff [55] recommend that policies and research relating to retirement should ‘focus on the household, not the individual; consider retirement as an often messy and disrupted process and not a discrete event; and understand that retirement may mean very different things for women and men’. Within this review, two papers [29, 31] supported this, outlining when looking at female retirement, there is a greater need to account for partner status.

#### Workplace factors

This review has highlighted a number of organisational barriers and facilitators to EWL, both physical and psychological, with work-related factors reported in nine out of the 15 papers reviewed [29, 30, 32, 34–36, 38–40]. The key physical barriers included those associated with manual work, organisation size (with larger organisations cited as being less accommodating) and lack of training and development opportunities for older workers. A previous review in the UK [48] found that physical work demands led to ill health, or worsened existing conditions; highlighting that currently work-based risk assessments are based on a younger working population. This review supports previous research suggesting psychological barriers to EWL include low job control, lack of flexibility and choice, organisational culture and climate, including job satisfaction, with those in more dissatisfying jobs significantly more likely to retire, relative to satisfied workers [60] and that high work pressure; insufficient use of skills and knowledge impact on ability to EWL [34].

Further barriers found in the current review support evidence from previous reviews, which include the barriers as follows: negative or ageist attitudes, particularly in respect of the ability to deal with the demands of a fast-paced and competitive workplace [32]; and negative perceptions of self [61]. Our review also corroborates research across the EU showing that age related discrimination has been increasing. In fact, 58% of European respondents suggest that age discrimination based on age stereotypes was widespread in the work place [62]. However, regarding age-related stereotypes, research within the USA has found that those relating to the productivity and motivational levels of older workers are in fact unwarranted, suggesting a dissonance among employers’ beliefs about older workers [63]. Importantly though, stereotypes in the workplace have been found to be malleable and therefore changeable so there is scope for change [64]. Similarly, van der Heijden et al. [65] found that across Europe, there were negative effects of dissimilarity in the supervisor-employee relationship, whereby a high-quality relationship of this kind mediated any potentially harmful effect. This suggests that the supervisor-employee relationship might be a particularly useful way of mediating any of the problems highlighted through potential ageism.

To facilitate EWL, Altman [53] urges the eradication of discrimination during the recruitment process, where often wording, such as ‘recent graduate’ or ‘energetic’ discriminate against older workers applying, calling on the government to fund a major national ‘age confident’ project and communication campaign, modelled on the success of ‘disability confident’. This campaign should highlight the business and economic case for ensuring older workers participate in the labour force and aim to break down barriers, such as negative stereotypes. A similar national intervention programme has been developed in The Netherlands aimed at targeting age stereotypes within the workplace around older workers, as well as increasing employability of older employees and improving business performance [66].

Workplace factors found to be facilitative of EWL in the current review related to flexible working conditions, working in smaller organisations, effective balancing between resources and demands, the social role of work and voluntary work, as well as making a positive contribution, but also a range of other factors, which are summarised in a number of previous reviews [16, 45]. To enable successful interventions monitoring demographic patterns of sickness absence and presenteeism (particularly for the 40+ age group) is recommended, which would enable epidemiological data on known risk factors to be gathered, and vulnerable groups identified by job role [16, 44]. Monitoring should be undertaken alongside a range of primary, secondary and tertiary interventions, including sustainable health promotion, improving the physical work environment (reduce physical loads, rehabilitative adaptations lighting, noise levels and thermal environment), flexible working (discussed above) alongside looking at work patterns, e.g. shift work, where extended recovery periods should be offered to 45–50+ year olds and exposure to long shifts minimised. In this regard, research from the USA ‘Age and Generations Study’ found that flexible workplaces are ‘all the rage’ and over 78% of respondents felt that flexible practices contributed to their success to a great extent [67].

#### Financial security and pension arrangements

Women have been disadvantaged in recent years by changes to the state pension age and the harmonisation of state pension age for men and women, meaning women are forced to work for longer in many European countries including the UK [68]. In fact, in European countries such as France, there have been mass protests in refute of the rise in retirement age for both genders, with millions taking part in mass demonstrations. There is also a global phenomenon with women across the world being disadvantaged in the workplace [69], and the risk of poverty in old age is greater for women than men [70]. Income has a major impact on retirement choices, with Bloom [71] stating that ‘the increase in real wages has been the main determinant of the long-term decline in the retirement age in industrialised countries’. For routine workers, with lower pension entitlements and limited access to firm-sponsored retirement plans, they tend to retire later, except in the case of disability.

Gender has been shown to be a significant predictor of the financial reasons for EWL, and women were more likely to take on bridge employment (any paid work after an individual retires or starts receiving a pension) for financial reasons than men [72]. This is because part-time jobs are associated with an ‘hourly wage penalty’ and contribute to the risk of being poor [52]. As a result, for women in particular, any lack of opportunities to take on more flexible bridge employment could act as a potential barrier to EWL. Similarly, the short-term effect of pension reform in Europe for 50–64 year olds has found that, due to the differing lengths of time that women and men spend in employment and the more stable career paths of males compared to females, pension reforms have resulted in a short-term positive impact on female participation, compared to a modest reduction in male participation [18].

Addressing the gender pay gap could have a positive impact on the social class of women in Europe, which may reduce their likelihood of working in routine, manual jobs, known to be related to involuntary retirement through disability. Fasang [73] emphasises that ‘social policies that address women’s income in retirement need to orchestrate different policy areas that affect women to a greater or lesser degree at different stages of their life course’. Similarly, policies aimed at prolonging work life may need to consider care responsibilities [74] because globally ‘work-family balance was the top work-related issue for women’ [69].

## Conclusions

Demographic changes mean that fuller participation in the labour market through EWL is essential; however, older women are an ‘under-represented’ group. As a result, this review has explored the key barriers and facilitators to EWL in Europe and sought to understand *why* older women, in particular, are under-represented and to identify the gaps in the literature.

The reasons for exiting the labour market are complex and they accumulate over the life course, impacting differently on men and women. Negative impacts of social class on health reduce the opportunity for ‘healthy ageing’, leading to a strong gradient in disability by occupational class, with routine and manual workers more likely to be impacted. Inextricably linked to these socio-economic factors are the impacts of income on retirement choices, and unsurprisingly routine workers, with lower pension entitlements, have fewer options and tend to retire later, unless they have been pushed out through ill health. Within the workplace, physical workload and psychological factors shape retirement choices, particularly in respect of levels of job control, manual work and attitudes towards older workers. Social factors also impact on retirement choices, with push factors associated with wanting to do things outside of work and wanting to enjoy life now, influenced by marital status, and domestic responsibilities.

As the reasons for EWL accumulate over the life course and health is the largest contributor to early labour market exit, it is important to focus on the determinants of healthy ageing across the life course (including those relating to women’s health needs) rather than solely focusing on ‘older workers’. In this respect, the literature has highlighted a key gap in the evidence in respect of workplace interventions. The workplace is a key setting for health promotion, and interventions to provide ‘good work’ that promotes health should be a key priority for public health professionals and employers—although more research and intervention evaluations are needed in this area. Improving health in the workplace has the potential to have a greater impact on routine and manual workers and women; two of the groups that are at increased risk of retiring early.

In respect of income and policy changes to statutory pensions, Radl [41] warns that ‘the more the age of retirement per se matters for old-age pension eligibility and the calculation of benefits, the more detrimental it is for working class individuals who still frequently retire involuntarily’, particularly due to ill health. Policy changes relating to increasing the statutory pension age have been found to be more effective for women, although the reasons for this appear to relate more to the fact that this group are less likely to be able to afford to retire early. This suggests that gender-specific policies, particularly those aimed at addressing the disadvantaged class position that women find themselves in should be considered. Policies that should be explored further include those aimed at maternity benefits, parental leave and good quality flexible work (and working arrangements) that facilitate caring responsibilities across the life course, *without* disadvantaging women’s careers. Again, further research is needed; however, given that health is linked to socio-economic position and EWL, policies aimed at reducing the negative impact of discontinuous employment careers are likely to have a positive impact on women, their health, and their ability to extend their working lives.

This review had underlined the task for policy makers and employers in terms of keeping the workforce not only active in the labour market but also healthy. Many women are now forced to extend their working lives due to policy changes in respect of state pension entitlements, such as the case in the UK. Workplaces must adapt to the needs of older women. To do this, special attention should be paid to the role of individual level factors such as caring responsibilities and macro level factors such as opportunities for training and development and challenging unhelpful social norms and stereotypes. Finally, for women to remain healthier in the workforce for longer, it is essential that their unique needs are protected and the workplace is both aware of these needs and, in response, is flexible in its approach to extending working life.

## Abbreviations

EEA: European Economic Area
EU: European Union
EWL: Extending working life
UK: United Kingdom

## References

[1] United Nations (2013). World Population Ageing 2013.

[2] Marmot M, Allen J, Goldblatt P, Boyce T, McNeish D, Grady M (2010). Fair society, healthy lives: strategic review of health inequalities in England post-2010.

[3] Eurostat. Mortality and life expectancy statistics. 2016. Available from: http://ec.europa.eu/eurostat/statistics-explained/index.php/Mortality_and_life_expectancy_statistics. Accessed 17 Jul 2016.

[4] European Commission. The 2012 ageing report: economic and budgetary projections for the 27 EU member states (2010−2060), in European Commission. 2012. p. 2012. Available from: http://ec.europa.eu/economy_finance/publications/european_economy/2012/pdf/ee-2012-2_en.pdf. Accessed 20 Aug 2016.

[5] Department for Work and Pensions (2014). Pensions Act 2014.

[6] Finnish Centre for Pensions. Retirement ages in member states. 2016. Available from: http://www.etk.fi/en/the-pension-system-2/the-pension-system/international-comparison/retirement-ages/. Accessed 10 Aug 2016.

[7] Eurofound. The role of governments and social partners in keeping older workers in the labour market. 2013. Available from: http://www.eurofound.europa.eu/publications/report/2013/working-conditions-labour-market-social-policies/role-of-governments-and-social-partners-in-keeping-older-workers-in-the-labour-market. Accessed 20 Aug 2016.

[8] Eurostat. Gender pay gap statistics. 2016; Available from: http://ec.europa.eu/eurostat/statistics-explained/index.php/Gender_pay_gap_statistics#Further_Eurostat_information. Accessed 20 Aug 2016.

[9] Waddell G, Burton AK (2006). Is work good for your health and well-being?.

[10] World Health Organization. Ottawa charter for health promotion. 1986: Geneva.

[11] Behncke S (2012). Does retirement trigger ill health?. Health Econ.

[12] Kuhn A, Wuellrich J-P, Zweimüller J (2010). Fatal attraction? Access to early retirement and mortality.

[13] Sahlgren GH. Work Longer, Live Healthier: The relationship between economic activity, health and government policy. 2013, Institute of Economic Affairs. Available from:http://iea.org.uk/sites/default/files/publications/files/Work%20Longer,%20Live_Healthier.pdf.

[14] van der Heide I, van Rijn RM, Robroek SJ, Burdorf A, Proper KI (2013). Is retirement good for your health? A systematic review of longitudinal studies. BMC Public Health.

[15] Europe2020. Female labour market participation. Available from: http://ec.europa.eu/europe2020/pdf/themes/2015/labour_market_participation_women_20151126.pdf. Accessed 27 Jul 2015.

[16] Ilmarinen J (2012). Promoting active ageing in the workplace. European Agency for Safety and Health at Work.

[17] Eurofound. Employment trends and policies for older workers in the recession. 2012. Available from: http://www.eurofound.europa.eu/sites/default/files/ef_publication/field_ef_document/ef1235en.pdf. Accessed 27 Jul 2015.

[18] Arpaia A, Dybczak K, Pierini F (2009). Assessing the short-term impact of pension reforms on older workers’ participation rates in the EU: a diff-in-diff approach.

[19] Eurostat. Employment rate of people aged 20 to 64 in the EU up to 69.2% in 2014: new peaks for women and those aged 55–64. 2015: Eurostat newsrelease. Available from: http://ec.europa.eu/eurostat/documents/2995521/6823708/3-07052015-AP-EN.pdf/7e507ea0-43c7-452f-8e6a-b479c89d2bd6.

[20] Penfold M, Foxton F (2015). Participation rates in the UK-2014-3. Older people.

[21] Kadefors R. Costs and benefits of best agers employment. WP3 Activity 2. 2011, University of Gothenburg. Available from: http://www.best-agers-project.eu/Portals/18/WP3/Best%20Agers_WP3_Costs_and_benefits.pdf.

[22] Organisation for Economic Co-operation and Development (2012). Closing the gender gap: act now.

[23] Eurostat. Labour force survey statistics—transition from work to retirement. 2012. Available from: http://ec.europa.eu/eurostat/statistics-explained/index.php/Labour_force_survey_statistics_-_transition_from_work_to_retirement. Accessed 15 Jul 2016.

[24] Arksey H, O’Malley L (2005). Scoping studies: towards a methodological framework. Int J Soc Res Methodol.

[25] Edwards S, Brettle A (2016). The value of trained library and information professionals.

[26] Slater L. Product review: PubMed PubReMiner. Journal of the Canadian Health Libraries Association/Journal de l’Association des bibliothèques de la santé du Canada 2014;33:106-107.

[27] Osei-Kwasi HA, Nicolaou M, Powell K, Terragni L, Maes L, Stronks K (2016). Systematic mapping review of the factors influencing dietary behaviour in ethnic minority groups living in Europe: a DEDIPAC study. Int J Behav Nutr Phys Act.

[28] Solem PE, Syse A, Furunes T, Mykletun RJ, De Lange A, Schaufeli W (2016). To leave or not to leave: retirement intentions and retirement behaviour. Ageing Soc.

[29] Nicolaisen M, Thorsen K, Eriksen SH (2012). Jump into the Void? Factors related to a preferred retirement age: gender, social interests, and leisure activities. Int J Aging Hum Dev.

[30] Loretto W, White P (2006). Work, more work and retirement: older workers’ perspectives. Soc Policy Soc.

[31] Rice NE, Lang IA, Henley W, Melzer D (2011). Common health predictors of early retirement: findings from the English Longitudinal Study of Ageing. Age Ageing.

[32] Porcellato L, Carmichael F, Hulme C, Ingham B, Prashar A (2010). Giving older workers a voice: constraints on the employment of older people in the North West of England. Work Employ Soc.

[33] Brown P, Vickerstaff S (2011). Health subjectivities and labor market participation pessimism and older workers’ attitudes and narratives around retirement in the United Kingdom. Res Aging.

[34] Reeuwijk KG, de Wind A, Westerman MJ, Ybema JF, van der Beek AJ, Geuskens GA (2013). ‘All those things together made me retire’: qualitative study on early retirement among Dutch employees. BMC Public Health.

[35] van Solinge H, Henkens K (2014). Work-related factors as predictors in the retirement decision-making process of older workers in the Netherlands. Ageing Soc.

[36] Thorsen S, Rugulies R, Løngaard K, Borg V, Thielen K, Bjorner JB (2012). The association between psychosocial work environment, attitudes towards older workers (ageism) and planned retirement. Int Arch Occup Environ Health.

[37] Tüchsen F, Christensen KB, Lund T, Feveile H (2008). A 15-year prospective study of shift work and disability pension. Occup Environ Med.

[38] Larsen M (2008). Does quality of work life affect men and women’s retirement planning differently?. Appl Res Qual Life.

[39] Micheel, F., J. Roloff, and I. Wickenheiser. The impact of socioeconomic characteristics on older employees’ willingness to continue working in retirement age. Comp Popul Stud. 2011;35(4)869-902.

[40] Hofacker D, Schroder H, Li Y, Flynn M (2016). Trends and determinants of work-retirement transitions under changing institutional conditions: Germany, England and Japan compared. J Soc Policy.

[41] Radl J (2013). Labour market exit and social stratification in Western Europe: the effects of social class and gender on the timing of retirement. Eur Sociol Rev.

[42] De Preter H, Van Looy D, Mortelmans D (2013). Individual and institutional push and pull factors as predictors of retirement timing in Europe: a multilevel analysis. J Aging Stud.

[43] Phillipson C, Smith A (2005). Extending working life: a review of the research literature.

[44] Weyman A, Meadows P, Buckingham A (2013). Extending working life audit of research relating to impacts on NHS employees. NHS Working Longer Review.

[45] Boot CR, van den Heuvel SG, Bültmann U, de Boer AG, Koppes LL, van der Beek AJ (2013). Work adjustments in a representative sample of employees with a chronic disease in the Netherlands. J Occup Rehabil.

[46] Lahelma E, Uusitalo H, Martikainen P (2012). Longer work careers through tackling socioeconomic inequalities in disability retirement. Eur J Pub Health.

[47] World Health Organization. Healthy workplaces: a model for action—for employers, workers, policy-makers and practitioners. 2010. Available from: http://www.who.int/occupational_health/publications/healthy_workplaces_model.pdf. Accessed 20 Aug 2015.

[48] Buckle P (2015). Workplace infrastructure. Future of ageing: evidence review. Foresight.

[49] Cloostermans L, Bekkers MB, Uiters E, Proper KI (2015). The effectiveness of interventions for ageing workers on (early) retirement, work ability and productivity: a systematic review. Int Arch Occup Environ Health.

[50] McDermott H, Kazi A, Munir F, Haslam C (2010). Developing occupational health services for active age management. Occup Med.

[51] Pitt-Catsouphes M, James JB, Matz-Costa C (2015). Workplace-based health and wellness programs: the intersection of aging, work, and health. The Gerontologist.

[52] van Lancker W, Corluy V, Horemans J, Marchal S, Vinck J, Perrons D (2015). Main causes of female poverty. Compliation of in-depth analyses workshop, 30th March 2015.

[53] Altman R (2015). A new vision for older workers: retain, retrain, recruit.

[54] Shacklock K, Brunetto Y, Nelson S (2009). The different variables that affect older males’ and females’ intentions to continue working. Asia Pac J Hum Resour.

[55] Loretto W, Vickerstaff S (2013). The domestic and gendered context for retirement. Hum Relat.

[56] Office for National Statistics. Full report—women in the labour market. 2008: London.

[57] Fernández-Ballesteros R, Zamarrón MD, Díez-Nicolás J, López-Bravo MD, Molina MÁ, Schettini R (2011). Productivity in old age. Res Aging.

[58] Irving P, Steels J, Hall N (2005). Factors affecting the labour market participation of older workers: qualitative research.

[59] Aranki, T. and C. Macchiarelli. Employment duration and shifts into retirement in the EU. 2013, LSE ‘Europe in Question’ Discussion Paper Series. Available from: http://www.lse.ac.uk/europeanInstitute/LEQS%20Discussion%20Paper%20Series/LEQSPaper58.pdf. Accessed 20 Aug 2016.

[60] Clark AE, Mavromaras K, Wei Z (2014). Happy to stay: job satisfaction and retirement.

[61] Benjamin K, Wilson S (2005). Facts and misconceptions about age, health status and employability.

[62] Eurobarometer 317 (2009). Discrimination in the EU in 2009.

[63] Ng TWH, Feldman DC (2012). Evaluating six common stereotypes about older workers with meta-analytical data. Pers Psychol.

[64] Molden DC, Dweck CS (2006). Finding “meaning” in psychology: a lay theories approach to self-regulation, social perception, and social development. Am Psychol.

[65] van Der Heijden BI, Scholarios D, Van Der Schoot E, Jedrzejowicz P, Bozionelos N, Epitropaki O (2010). Supervisor-subordinate age dissimilarity and performance ratings: the buffering effects of supervisory relationship and practice. Int J Aging Hum Dev.

[66] van Selm M, van der Heijden BI (2013). Communicating employability enhancement throughout the life-span: a national intervention program aimed at combating age-related stereotypes at the workplace. Educ Gerontol.

[67] Pitt-Catsouphes M, Matz-Costa C, Bessen M, T.S. Centre (2009). Workplace flexibility: findings from the age & generations study. Aging and Work.

[68] Eurofound. Reform of old age pension and retirement systems in the EU. Eurwork: European Observatory of Working Life 2015; Available from: http://www.eurofound.europa.eu/observatories/eurwork/articles/working-conditions-industrial-relations-law-and-regulation/reforms-of-old-age-pensions-and-retirement-systems-q1-2015. Accessed 20 Aug 2016.

[69] International Labour Organisation (2016). Women in Work- Trends 2016.

[70] Woetzel J, A Madgavkar, K Ellingrud, E Labaye, S Devillard, E Kutcher et al. The power of parity: how advancing women’s equality can add $12 trillion to global growth. 2015, McKinsey Global Institute. Available from: http://www.mckinsey.com/global-themes/employment-and-growth/how-advancing-womens-equality-can-add-12-trillion-to-global-growth.

[71] Bloom DE, D Canning, M Moore. A theory of retirement. USA: National Bureau of Economic Research; 2007.

[72] Vignoli D, ML Tanturri, F Acciai. Home bitter home? Gender, living arrangements, and the exclusion from home-ownership among older Europeans. Italy: Universita’degli Studi di Firenze, Dipartimento di Statistica, Informatica, Applicazioni “ G. Parenti”; 2014.

[73] Fasang AE, Aisenbrey S, Schömann K (2013). Women’s retirement income in Germany and Britain. Eur Sociol Rev.

[74] Lumsdaine RL, Vermeer SJ (2015). Retirement timing of women and the role of care responsibilities for grandchildren. Demography.

